# Effect of primary lesions in cytoskeleton proteins on red cell membrane stability in patients with hereditary spherocytosis

**DOI:** 10.3389/fphys.2022.949044

**Published:** 2022-08-12

**Authors:** Cristina Vercellati, Anna Paola Marcello, Bruno Fattizzo, Anna Zaninoni, Agostino Seresini, Wilma Barcellini, Paola Bianchi, Elisa Fermo

**Affiliations:** ^1^ Fondazione IRCCS Ca’ Granda Ospedale Maggiore Policlinico Milano—UOC Ematologia, UOS Fisiopatologia Delle Anemie, Milan, Italy; ^2^ Fondazione IRCCS Ca’ Granda Ospedale Maggiore Policlinico Milano—UOC Laboratorio Centrale, UOS Laboratorio Genetica Medica, Milan, Italy

**Keywords:** hereditary spherocytosis, hemolytic anemias, cytoskeleton, RBC membrane defects, NGS

## Abstract

We investigated by targeted next generation sequencing the genetic bases of hereditary spherocytosis in 25 patients and compared the molecular results with the biochemical lesion of RBC membrane obtained by SDS-PAGE analysis. The HS diagnosis was based on available guidelines for diagnosis of congenital hemolytic anemia, and patients were selected because of atypical clinical presentation or intra-family variability, or because presented discrepancies between laboratory investigation and biochemical findings. In all patients but 5 we identified pathogenic variants in *SPTA1, SPTB, ANK1, SLC4A1, EPB42* genes able to justify the clinical phenotype. Interestingly, a correspondence between the biochemical lesion and the molecular defect was identified in only 11/25 cases, mostly with band 3 deficiency due to *SLC4A1* mutations. Most of the mutations in *SPTB* and *ANK1* gene didn’t hesitate in abnormalities of RBC membrane protein; conversely, in two cases the molecular lesion didn’t correspond to the biochemical defect, suggesting that a mutation in a specific cytoskeleton protein may result in a more complex RBC membrane damage or suffering. Finally, in two cases the HS diagnosis was maintained despite absence of both protein defect and molecular lesion, basing on clinical and family history, and on presence of clear laboratory markers of HS. The study revealed complex relationships between the primary molecular lesion and the final effect in the RBC membrane cytoskeleton, and further underlines the concept that there is not a unique approach to the diagnosis of HS.

## Introduction

Hereditary Spherocytosis (HS) is considered to be the most common congenital hemolytic anemia, with an estimated prevalence of about 1:2,000 in the Northern European population (Gallagher 2005; Perrotta et al., 2008; Bianchi and Mohandas, 2016). Seventy-five percent of the cases have an autosomal dominant inheritance and the remaining twenty five percent are inherited in an autosomal recessive way or due to *de novo* mutations. The main clinical features are hemolytic anemia of variable degree from compensated to severe, sometimes requiring exchange transfusion at birth and/or repeated blood transfusions, variable jaundice, splenomegaly, and cholelithiasis (Kalfa, 2021).

The clinical variability reflects the molecular complexity of this disorder due to mutations in different genes codifying for proteins directly involved in the cytoskeleton structure (*SPTA1* and *SPTB* codifying for alpha- and beta-spectrin) or in the attachment of cytoskeleton to the overlying lipid bilayer (*ANK1, SLC4A1, EPB42,* ankyrin, band 3, and protein 4.2) (Narla and Mohandas, 2017; Iolascon et al., 2019; Kalfa 2021). Abnormalities in such membrane components lead to the release of microvesicles and the formation of spheroidal, osmotically fragile red blood cells (RBCs) that are selectively trapped in the spleen and that survive almost normally after splenectomy. Given the rarity and the wide clinical heterogeneity, the diagnosis of HS can be challenging (Fermo et al., 2021a). The diagnostic workflow is based on clinical and family history, biochemical hemolysis parameters, RBC peripheral blood examination and functional tests investigating osmotic fragility which is typically reduced in spherical-shaped erythrocytes. Other diagnostic tools with high sensitivity and specificity include eosin 5-maleimide (EMA)-binding test (King et al., 2000; Bianchi et al., 2012), and ektacytometry, in particular the new generation ektacytometer laser-assisted optical rotational cell analyzer (LoRRca) Osmoscan, that however is available only in specialized laboratories (Da Costa et al., 2016; Lazarova et al., 2017; Zaninoni et al., 2018).

The defective RBC membrane proteins identified by sodium dodecyl sulphate-polyacrylamide gel electrophoresis (SDS-PAGE), have been considered for many years a confirmatory test for membrane defects in HS; however, it is known that a in about 10% of the patients, the protein deficiency remains unclassified by this technique (Mariani et al., 2008; Bianchi et al., 2012). The development of next generation sequencing (NGS) techniques and targeted-NGS panels may be useful to unravel complex cases, since the genes encoding for RBC membrane proteins are now usually included in the targeted panels specifically designed for the diagnosis of hereditary hemolytic anemias; this resulted in a rapidly growing knowledge of the molecular basis of HS and of the genotype phenotype correlation (He et al., 2017; Russo et al., 2018; van Vuren et al., 2019; Xue et al., 2019; Bianchi et al., 2020; Qin et al., 2020; Fermo et al., 2021b; Vives-Corrons et al., 2021). However, few data are available regarding the direct consequences of the various genetic defects on the assembly and dynamic interactions of the RBC cytoskeleton. To investigate these aspects, we analyzed by t-NGS the genetic bases of hereditary spherocytosis in 25 patients, and compared the molecular results with the biochemical lesion obtained by SDS-PAGE analysis.

## Materials and methods

### Patients

Twenty-five HS patients (14 females and 11 males; median age 17 years; range 3 months–60 years) belonging to 23 unrelated families were investigated. Patients were selected because of atypical clinical presentation or intra-family variability, or presence of discrepancies between laboratory investigation and biochemical findings. Peripheral blood was collected from patients during diagnostic procedures after obtaining informed consent and approval from the institutional human ethics committee, in accordance with the Helsinki international ethical standards on human experimentation. The great majority of samples were collected at our institute; samples of other centers were shipped maintaining a temperature of 4°C and always processed within 24 h from collection. None of the patients had been transfused within the 3 months preceding the study. All patients underwent complete blood counts (Automatic LH-750, Beckman Coulter, Brea, CA), hemolysis markers, iron status (Integra 800, Roche, Mannheim, Germany), RBC morphology evaluation and osmotic fragility tests (NaCl osmotic fragility test on both fresh and incubated blood, glycerol lysis test (GLT), acidified glycerol lysis test (AGLT), Pink test). In addition, EMA-binding test (Bianchi et al., 2012) and assessment of erythrocyte deformability by laser-assisted optical rotational cell analyser (LoRRca MaxSis; Mechatronics, Zwaag, the Netherlands) were performed (Zaninoni et al., 2018).

HS was diagnosed on the basis of clinical and laboratory signs of chronic hemolysis, presence of spherocytes in peripheral blood smear examination, positivity of at least one RBC fragility test and EMA-binding test and LoRRca Osmoscan, family history of HS if any, and exclusion of other causes of secondary hemolysis in agreement with diagnostic recommendations by King *et al* (2015). The diagnostic workflow is summarized in Supplementary Figure S1.

### Analysis of red cell membrane proteins

Red cell ghosts were prepared within 24 h of blood collection using the method of Dodge et al (1963) with the following modifications: whole blood was collected in EDTA and centrifuged at 1,000 g for 10 min, plasma and buffy-coat were removed equilibrated with phosphate-buffered saline [154 mM NaCl, 10 mM phosphate buffer, 0.1 mM phenylmethylsulphonyl fluoride (PMSF), pH 7.4]. The lysis buffer was made of 5 mM phosphate buffer, 1 mM EDTA, 0.5 mM PMSF, pH 7.4. Free hemoglobin ghosts were stored frozen in aliquots after addition of 1% SDS and 5 mM N-ethyl-maleimide. Red cell membrane proteins were analyzed, within 15 days of preparation of the ghosts, by SDS-PAGE using a 3.5%–17% exponential gradient of acrylamide according to Fairbanks et al (1971) and the discontinuous buffer system of Laemmli (1970) with an acrylamide linear gradient from 6% to 14% (Mariani et al., 2008). Each patient and control sample was loaded at least four times on each gel and the average densitometric reading from Coomassie blue stained gels was considered (GS-800 Calibrated Densitometer, Bio-Rad). Quantitation of bands was expressed as ratios to band 3. Total spectrin (alpha + beta chains) and ankyrin deficiencies were defined as a ratio to band 3 below the lower limit of the reference range calculated from 100 normal subjects (reference ranges: spectrin/band 3: 0.95–1.17, ankyrin/band 3: 0.14–0.21). Band 3 deficiency was defined as a spectrin/band 3 ratio higher than the upper limit of the reference range (see above). Protein 4.2 deficiency was defined as an isolated, substantial decrease of the protein 4.2/band 3 ratio (<50% of the lower limit of the reference range: 0.15–0.20). The HS patients were grouped according to the type of membrane defect: band 3, spectrin, ankyrin, or UND (undetectable defect), as assessed by SDS-PAGE.

### Next generation sequencing analysis

Genomic DNA was extracted from peripheral blood using standard manual methods and quantified by Nandrop One (Thermo Scientific, Italy). When available, relatives of affected cases were also enrolled to analyze allelic segregation and correctly assess the pathogenicity of each variant.

The DNA samples were analysed on an NGS-targeted panel containing 43 genes associated with congenital hemolytic anemia (Supplemetary Table S1) (Fermo et al., 2021b). Libraries were obtained by HaloPlexHS Target Enrichment System Kit (Agilent Technologies, Santa Clara, United States) and sequenced on a MiSeq platform (Illumina, San Diego, United States).

Sequencing reads were aligned against reference genome (UCSC hg19) and variants were called and annotated using the SureCall software (Agilent Technologies). Targeted filtering and annotation of protein-changing variants were performed using the wANNOVAR web tool (http://wannovar.wglab.org/).

Variants were assessed by mutation prediction and conservation programs including SIFT (http://sift.jcvi.org/), polyphen-2 (Polymorphism Phenotyping v2) (http://genetics.bwh.harvard.edu/pph2/) and MutationTaster (http://www.mutationtaster.org/), VarSome (https://varsome.com/); pathogenicity was evaluated according to the guidelines of American College of Medical Genetics and Genomics (ACMG) (Richards et al., 2015) using the online tool VarSome. (Kopanos et al., 2019).

Variants previously classified as pathogenic by databases such as ClinVar, HGMD, dbSNP, deleterious variants expected to produce truncated or abnormal protein, or splice site variants were considered as causatives, and confirmed by Sanger sequencing.

Nucleotide numbering reflects cDNA numbering with +1 corresponding to the A of ATG translation initiation codon in the reference sequence, according to the nomenclature for the description of sequence variants of Human Genome Variation Society (www.hgvs.org/mutnomen).

## Results

### Hematologic and biochemical results

Table 1 shows the hematologic and biochemical data of the 25 patients studied. Laboratory tests with higher sensitivity for HS diagnosis were: AGLT (positive in 21/25 patients analysed), Incubated NaCl osmotic fragility curve (positive in 17/24), EMA-binding test (23/24) and LoRRca ektacytometry (17/17 patients). Ten patients were positive to all the four tests, and ten were positive to three of them.

**TABLE 1:** Clinical and laboratory characteristics of the 25 HS patients at the time of diagnosis.

Pt	Age (yrs)	Sex	Family history	TX	Splenect (age)	Hb (g/dl)	Spherocytes (%)	Retics (X10^9^/L)	Unc bil. (mg/dL)	AGLT_50_ (%)	Osmotic fragility 37°C	LORRCA Osmoscan profile	Ema binding test (% reduction)	Biochemical defect at SDS- PAGE
1	7m	F	no	yes	no	9.6	1	n.a	n.a	520	increased	HS	16%	UND
2	24	M	yes	no	no	12	5	219	0.83	112	normal	HS	20%	UND
3	1	M	yes	no	no	9.8	7	269	1.93	89	increased	HS	35%	UND
4	3	F	no	no	no	11.4	12	113.7	2.12	72	increased	HS	33%	UND
5	49	F	yes	n.a	no	14.7	8	97.3	n.a	21	increased	HS	27%	UND
6	12	F	yes	no	no	10	18	435	2.67	82	increased	HS	28%	UND
7	39	M	yes	no	no	14.9	3	235	2.89	>900	normal	HS	26%	UND
8	45	M	no	no	no	14.1	13	259	1.61	>900	normal	HS	18%	UND
9	13	M	no	no	no	12.1	5	740	1.24	188	increased	HS	23%	band 3 (−19%) band 4.2 (−76%)
10	3m	F	yes	no	no	12.1	6	173	n.a	45	normal	HS	25%	band 3 (−18%)
11	15	M	yes	no	no	13.6	10	192	2.21	59	increased	n.a	38%	band 3 (−38%)
12	33	F	no	n.a	n.a	12.8	Nd	49	1.43	49	increased	n.a	27%	band 3 (−55%)
13	17	F	yes	yes	no	11	19	487	5.51	26	increased	HS	41%	band 3 (−29%)
14	3	F	no	n.a	n.a	n.a	n.a	n.a	n.a	170	normal	HS	34%	band 3 (−53%)
15§	23	M	yes	yes	yes (6)	13.6	5	178	3	37	increased	n.a	39%	band 3 (−64%)
16§	60	M	yes	no	no	12.3	14	183	1.5	41	slightly increased	n.a	n.a	band 3 (−23%)
17	48	F	yes	yes	no	10.2	4	248	2.33	111	normal	HS	18%	spectrin (−15%)
18	27	M	yes	n.a	n.a	14	10	292	6.88	43	increased	n.a	40%	spectrin (−11%)
19	36	F	no	no	no	10.5	9	312	nd	>900	slightly increased	HS	9%	spectrin (−21%)
20	24	F	no	no	no	10.3	15	409	3.57	33	increased	HS	30%	spectrin (−25%)
21	12	F	yes	no	yes (25)	12.9	6	66	0.6	78	increased	HS	18%	spectrin (−14%)
22	17	F	yes	no	no	10.9	9	554	n.a	77	slightly increased	n.a	26%	spectrin (−11%) band 4.2 (−30%)
23	8	M	yes	yes	yes (7)	9.2	50	439	3.94	14	increased	n.a	56%	spectrin (−68%) ankyrin (−56%)
24^a^	5	F	yes	yes	no	8.3	11	61.9	n.a	>900	normal	HS	13%	UND
25^a^	1	M	yes	yes	no	12.3	5	n.a	1.61	72	n.a	n.a	15%	ankyrin (−22%)
Ref. values						F 12–16 M 13.5–17.5		24–84	<0.8	>900	normal		<11%	

* 24–25 siblings; n. a. Not available; UND: undetected; TX, transfusions. § 15–16 son and father;

Median number of spherocytes, manually evaluated at two expert independent operators was 9% (range 1–50%), notably seven patients had 5% of spherocytes or less. In this series the number of spherocytes was not related with the severity of anemia, the type and entity of biochemical defect nor the kind of mutation.

All patients but one (case 19) had positive EMA-binding test, ranging from 13 to 56% in decrease in fluorescence (cut-off limit >11%). The diagnosis of HS in case 19 was done based on presence of mild hemolysis, presence of 9% spherocytes, LoRRca Osmoscan profile typical of HS and evidence of 21% decrease in spectrin content (interestingly, the patient showed a missense variant p. R870Q in *SLC4A1* gene, see later).

SDS-PAGE analysis of red cell membrane proteins revealed band 3 deficiency in 7 cases, spectrin in 5 cases, and ankyrin in one. Three patients showed combined defects (band 3/band 4.2, spectrin/band 4.2, and spectrin/ankyrin). The degree of band 3 deficiency was highly variable ranging from 18 to 64%, the decrease in spectrin content varied from 11 to 68%, or 11–25% not considering the combined spectrin/ankyrin deficiency). Protein 4.2 defect (ranging from 30 to 76%) was always found in combination with another protein defect. In 9 cases no defect was detectable despite clear diagnosis of HS. The absence of an evident membrane defects was independent from the clinical severity.

### Molecular investigations

**FIGURE 1:**
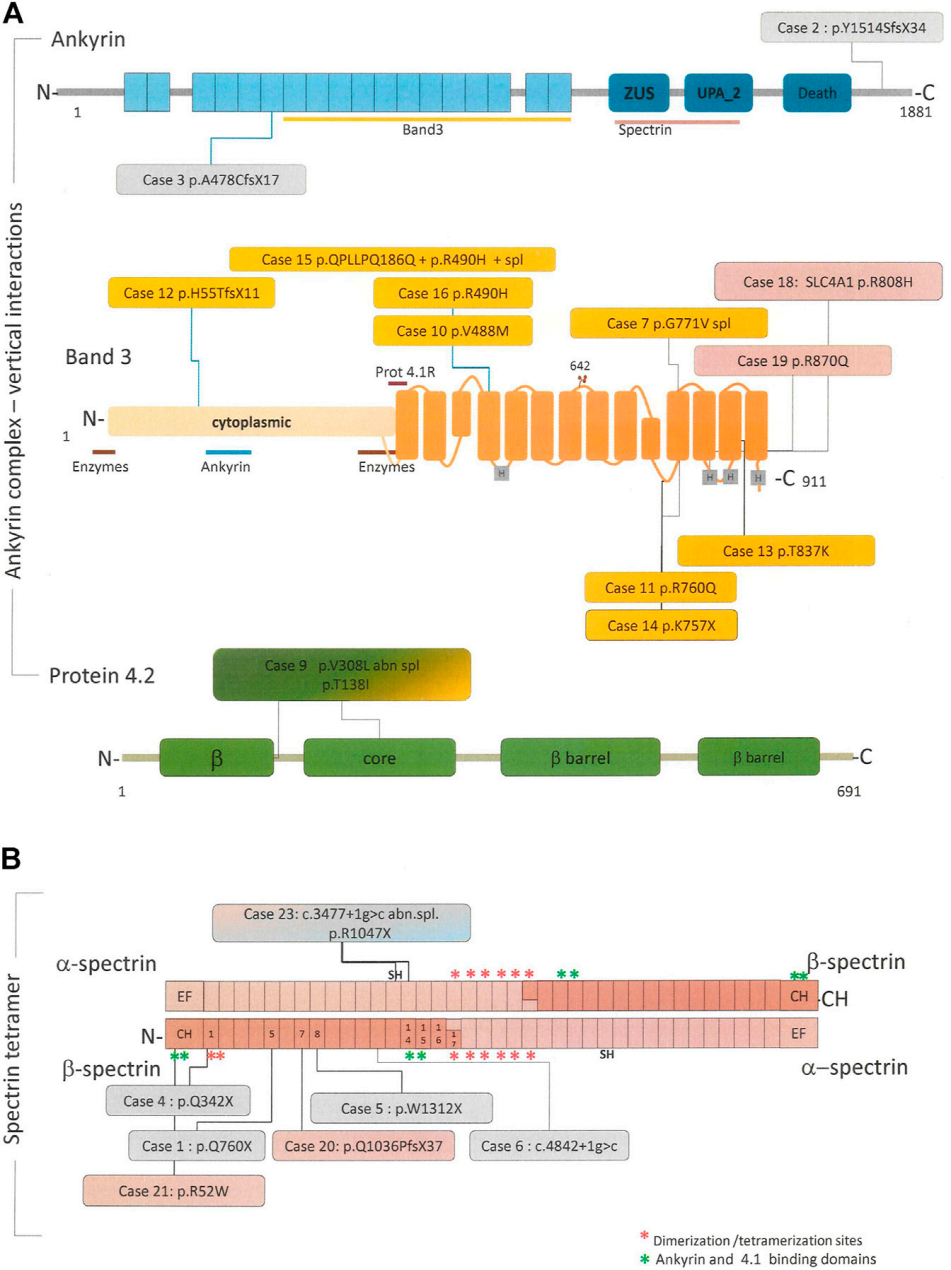
Linear schematic representation of the main proteins involved in the pathogenesis of HS, in particular ankyrin, band 4.2 and band 3 forming the “ankyrin complex “**(A)** and the alpha and beta spectrin tetramer **(B)**. When available, binding site for other cytoskeletal proteins or RBC enzyme are reported along the proteins. All the pathogenic variants identified in this study and their position in the respective proteins are reported in the colored boxes. Boxes colors correspond to the protein deficiency identified by SDS-PAGE analysis, in particular: pink = spectrin defect, orange = band 3 deficiency, light blue = ankyrin deficiency, green = 4.2 deficiency, and grey = no membrane defect.

To evaluate the concordance of the primary molecular lesion and the biochemical defect all patients underwent targeted-NGS. Table 2 shows the results of molecular analysis, and Figure 1 the distribution and kind of mutations on the corresponding proteins belonging to the ankyrin complex or spectrin tetramer respectively.

**TABLE 2:** Results of NGS analysis.

Pt	Biochemical defect	Gene (Ref.Sequence)	Mutation	Effect	Status	Pathogenicity	rs	Final diagnosis	Additional findings
1	UND	SPTB (NM_001355436.1)	c.2278C>T	p.Q760X	Het	5-P		SPTB	
2	UND	ANK1 (NM_000037.3)	c.4541delA	p.Y1514SfsX34	Het	5-P		ANK1	
3	UND	ANK1 (NM_000037.3)	c.1427_1430dupGTGC	p.A478CfsX17	het	5-P		ANK1	
4	UND	SPTB (NM_001355436.1)	c.1024C>T	p.Q342X	Het	5-P		SPTB	
5	UND	SPTB (NM_001355436.1)	c.3936G>A	p.W1312X	Het	5-P		SPTB	PKLR p.R486W
6	UND	*SPTB* (NM_001355436.1)	c.4842+1 g > *c*	Abn. Splicing	Het	5-P		SPTB	SPTA1^α−Lely^
7	UND	*SLC4A1* (NM_000342.3)	c.2312G>T	p.G771V/Abn. Splicing	Het	5-P		SLC4A1	
8	UND	UND	N.D.	N.D.	//	//		N.D.	SPTA1^α−Lely^ Hom
9	band 3 + band 4.2	*EPB42* (NM_000119.2)	c.922G>C	p.V308L/Abn. Splicing	Comp het	5-P	rs772330879	EPB42	
		*EPB42* (NM_000119.2)	c.413C>T	p.T138I	Comp het	4-LP			
10	band 3	*SLC4A1*(NM_000342.3)	c.1462G>A	p.V488M	Het	5-P	rs28931584	SLC4A1	
11	band 3	*SLC4A1*(NM_000342.3)	c.2279G>A	p.R760Q	Het	5-P	rs121912755	SLC4A1	SPTA1^α−Lely^
12	band 3	*SLC4A1*(NM_000342.3)	c.163delC	p.H55TfsX11	Het	5-P		SLC4A1	G6PD p.A335 T
13	band 3	*SLC4A1*(NM_000342.3)	c.2510C>A	p.T837K	Het	5-P		SLC4A1	
14	band 3	*SLC4A1*(NM_000342.3)	c.2269A>T	p.K757X	Het	5-P		SLC4A1	SPTA1^α−Lely^ *SEC23B* c.689 + 1g>a
15§	band 3	*SLC4A1*(NM_000342.3)	c.1469G>A	p.R490H	Comp het	5-P	rs1598299485	SLC4A1	SPTA1^α−Lely^
		*SLC4A1*(NM_000342.3)	c.558_572del	p.QPLLPQ186Q	Comp het	4-LP			
		*SLC4A1*(NM_000342.3)	c.1627–1 g > *t*	Abn. Splicing	Comp het	5-P			
16§	band 3	*SLC4A1*(NM_000342.3)	c.1469G>A	p.R490H	Het	5-P	rs1598299485	SLC4A1	SPTA1^α−Lely^
17	spectrin	N.D.	N.D.	//	//	//		N.D.	
18	spectrin	*SLC4A1*(NM_000342.3)	c.2423G>A	p.R808H	Het	5-P	rs866727908	SLC4A1	
		*ANK1* (NM_000037.3)	c.5600C>T	p.A1867V	Het	3-VUS	rs767580738		
19	spectrin	*SLC4A1*(NM_000342.3)	c.2609G>A	p.R870Q	Het	4-LP	rs746426065	SLC4A1	SPTA1^α−Lely^
20	spectrin	*SPTB* (NM_001355436.1)	c.3106dupC	p.Q1036PfsX37	Het	5-P		SPTB	SPTA1^α−Lely^
21	spectrin	*SPTB* (NM_001355436.1)	c.154C>T	p.R52W	Het	4-LP	rs1594796374	SPTB	SPTA1^α−Lely^
22	spectrin + band 4.2	N.D.	N.D.	//	//	//		N.D.	SPTA1^α−Lely^
23	spectrin + ankyrin	*SPTA1*(NM_003126.3)	c.3477+1 g > *c*	Abn. Splicing	Comp het	5-P		SPTA1	SPTA1^α−Lely^
		*SPTA1*(NM_003126.3)	c.3139C>T	p.R1047X	Comp het	5-P			
24*	UND	N.D.	N.D.	//	//	//		N.D.	
25*	ankyrin	ND.	N.D.	//	//	//		N.D.	

* 24-25 siblings. ND, not determined; UND, undetected; Het, heterozygous; Comp het, compound heterozygous; Hom, homozygous 5-P, pathogenic; 4-LP, likely pathogenic; 3-VUS, variant of uncertain significance § 15-16 son and father.

We identified 24 different pathogenic variants in *SPTA1, SPTB, ANK1, SLC4A1, EPB42,* all predicted as class 5 or 4 (pathogenic or likely pathogenic). Only a VUS variants (*ANK1* p. A1867V) was identified and reported as a possible modifier of clinical phenotype, because associated with a pathogenic variant in another gene (*SLC4A1*). No large deletions have been identified at CNVs analysis.

In all patients but 5 we identified pathogenic variants able to justify the clinical phenotype. Moreover, three cases presented pathogenic heterozygous variants in other genes associated with hemolytic anemias but not responsible for HS (case 12, female, *G6PD* p. A335T; case 5, *PKLR* p. R486W; case14, *SEC23B* c.689 + 1g>a). From patients history and clinical follow-up, it doesn’t seems that the concomitance of these variants contributed in worsening the clinical phenotype, although the possibility of any pathophysiological interaction remains to be determined. Finally, 11/25 patients presented the SPTA1^α−Lely^ polymorphic allele (*SPTA1*:c.6531–12C>T, p.? and p.L1858V), and none of them presented the SPTA1^α−LEPRA^ polymorphic allele (*SPTA1*:c.4339–99C>T).

Except for case 23, low allele expression polymorphisms didn’t worse the clinical phenotype, because not inherited *in trans* with another *SPTA1* variant.

Interestingly, a correspondence between the biochemical lesion and the molecular defect was identified in only 11/25 cases, mostly band 3 deficiency due to *SLC4A1* mutations (Table 2).

Out of the five cases with isolated spectrin defect (alpha + beta chains), only two cases had a corresponding *SPTB* defect mutation, two had a *SLC4A1* mutation and in the latter no molecular defect was found. Isolated ankyrin deficiency was present in only one case, where no mutation was detected in *ANK1* gene (case 25). Combined severe ankyrin/spectrin deficiency was attributed to two compound heterozygous *SPTA1* pathogenic variants, one of them associated with SPTA1^α−Lely^ polymorphic allele. Finally protein 4.2 deficiency was detected only in association with other protein deficiencies, in particular spectrin (no molecular variant identified) and band 3 (due to *EPB42* compound heterozygous mutations).

NGS analysis allowed identifying a molecular defect in seven out of the nine patients with undetectable defect at SDS-PAGE. Among them, three cases had *ANK1* mutations, three showed non sense variants in *SPTB* gene and one case had a new missense variant in *SLC4A1* gene (p.G771V) also resulting in an abnormal splicing.

**FIGURE 2:**
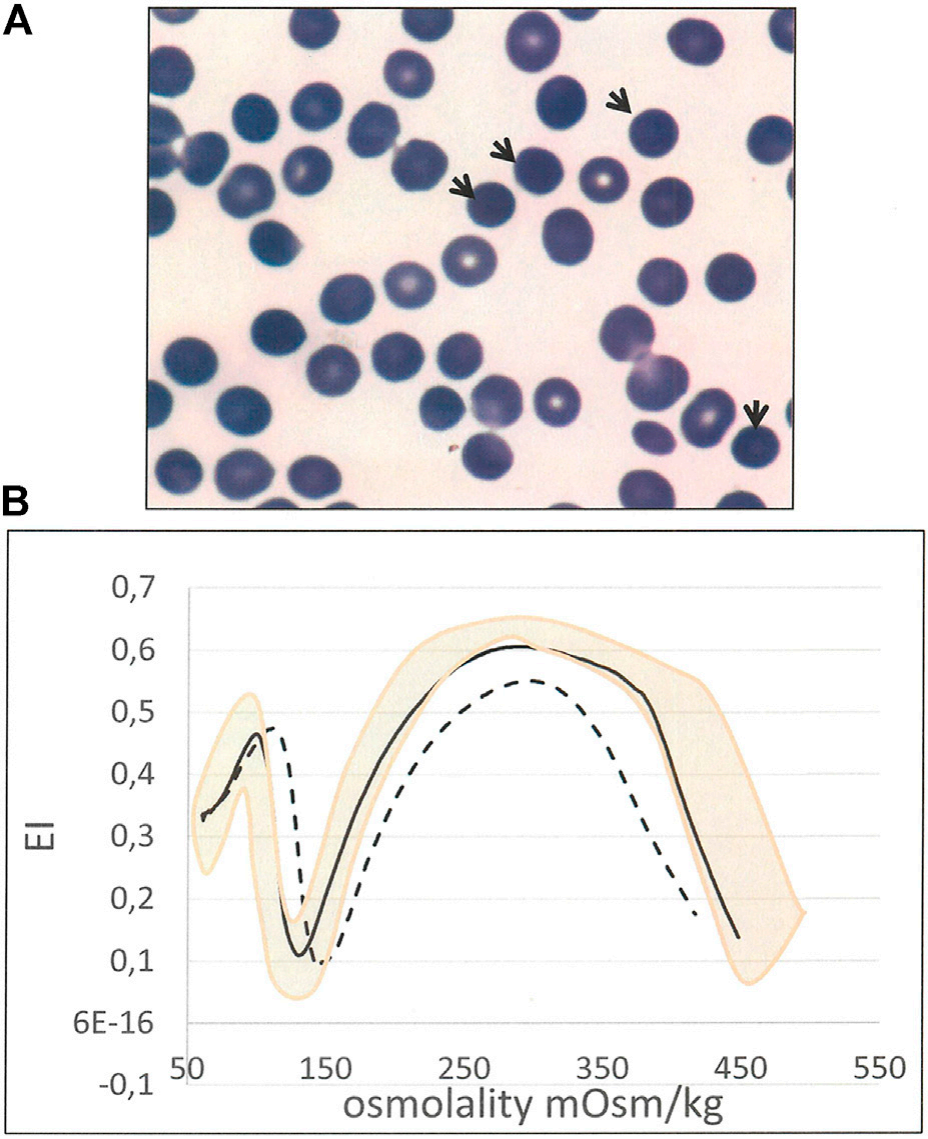
Case 8 **(A)** RBC peripheral blood film (100× magnification) showing spherocytes (arrows) **(B)** Ektacytometric analysis showing typical features of HS: increased Omin, decreased EI and Ohyper and AUC, in the patient (dotted line) compared with normal control (black line). Pink area represents the area covered by normal controls).

In two cases (8 and 24) the HS diagnosis was established in absence of both protein defect and molecular lesion. In case 8 it was based on history of compensated hemolysis and iron overload, presence of spherocytes in peripheral blood examination (13%), splenomegaly, cholelithiasis, positive EMA-binding (18% of reduction) and typical LoRRca Osmoscan profile. (Figure 2). Despite controversial laboratory results obtained in cases 24 and 25 - negative NGS and some borderline laboratory tests - the diagnosis of HS was maintained by clinicians basing on family history (a first cousin also affected with ankyrin deficiency), and complete clinical remission after splenectomy (performed in case 24 after this study).

## Discussion

The rapid evolution of NGS technologies and their even greater availability in routine application opened up exciting landscapes for diagnosis and study of molecular mechanisms of RBC disorders. However, the huge amount of data obtained makes the interpretation of the results and the identification of the pathogenic variant responsible for the diseases sometime difficult, often requiring additional functional analysis for variant validation. Consequently, NGS analysis is not always recommended as up-front investigation in the diagnosis of HS, or not recommended at all for patients with typical forms and positive family history (Bolton-Maggs et al., 2012; King et al., 2015).

Following the recommendations for diagnosis of HS and the more recent guidelines for the use of NGS in the diagnosis of rare inherited anaemias (Roy et al., 2022), in our study we limited the use of molecular investigations to 25 HS patients with atypical clinical presentation or intra-family variability, or patients who presented discrepancies between laboratory investigation and biochemical findings. Consequently, the results here reported cannot be considered representative of the entire HS population referred to our Centre (Mariani et al., 2008; Vercellati et al., 2022), and could not be in line with the distribution of biochemical and molecular abnormalities reported in different patients cohorts (Chonat et al., 2019; van Vuren et al., 2019; Wang et al., 2020; Vives-Corrons et al., 2021).

Despite that, by analyzing this particular series, we highlighted possible criticisms from both laboratory diagnostic workflow and molecular investigation in the diagnosis of HS. Moreover, the study provides novel insight in the pathophysiology of some RBC cytoskeletal defects and on the phenotypic effects of some variants on RBC membrane stability. Targeted-NGS analysis enabled to clarify the molecular basis of HS in 80% of the selected cases, a proportion higher than that identified by SDS-PAGE analysis (64% of the examined cases), but lower than the proportion of cases identified by laboratory investigations considered as gold standard for the diagnosis of HS (RBC peripheral blood morphology, EMA-binding test and ekacytometric analysis).

Surprisingly, a complete concordance of the membrane biochemical defect and primary molecular lesion was observed in 44% of the cases analyzed only. In a small proportion of cases (2/25) the primary defect didn’t hesitate in a deficiency of the corresponding protein: two missense *SLC4A1* mutations (p.R808H and p. R870Q, both located in the C-terminal part of the protein) resulted in spectrin deficiency, suggesting that the defect in a specific cytoskeleton protein may result in a more complex RBC membrane damage or suffering. There are a few data in literature on the correlation between genetics and membrane biochemical lesion (Jarolim et al., 1995; Bogardus et al., 2012; Iwase et al., 1998; Yawata et al., 2000; Da Costa et al,2013). Band 3 is a complex transmembrane molecule, with structural and transport functions (Bruce 2006; Jennings, 2021; Kalli and Reithmeier, 2022) and can be hypothesized that some *SLC4A1* mutations falling in a specific domain may disturb the underlying cytoskeleton, resulting in combined or secondary protein defects. Alpha-spectrin is overproduced (Hanspal and Palek, 1987) and as a consequence its deficiency is usually associated to homozygous of compound heterozygous *SPTA1* mutations causing significant quantitative defect. Moreover, it has been reported that spectrins may play a multitasking role in RBC physiology, cooperating in both the establishment and the maintenance of a diverse specialized plasma membrane domain, or being involved in an interface for signal transduction mediation, and in the interaction with membrane channels, adhesion molecules, receptors and transporters (Machnicka et al., 2014). As a consequence, it is possible that a genetic defect in different RBC proteins, may also result in a secondary spectrin defect as observed in Gardos channelopathy (Fermo et al., 2017) or some enzymopathies (Bianchi et al., 2003).

**FIGURE 3:**
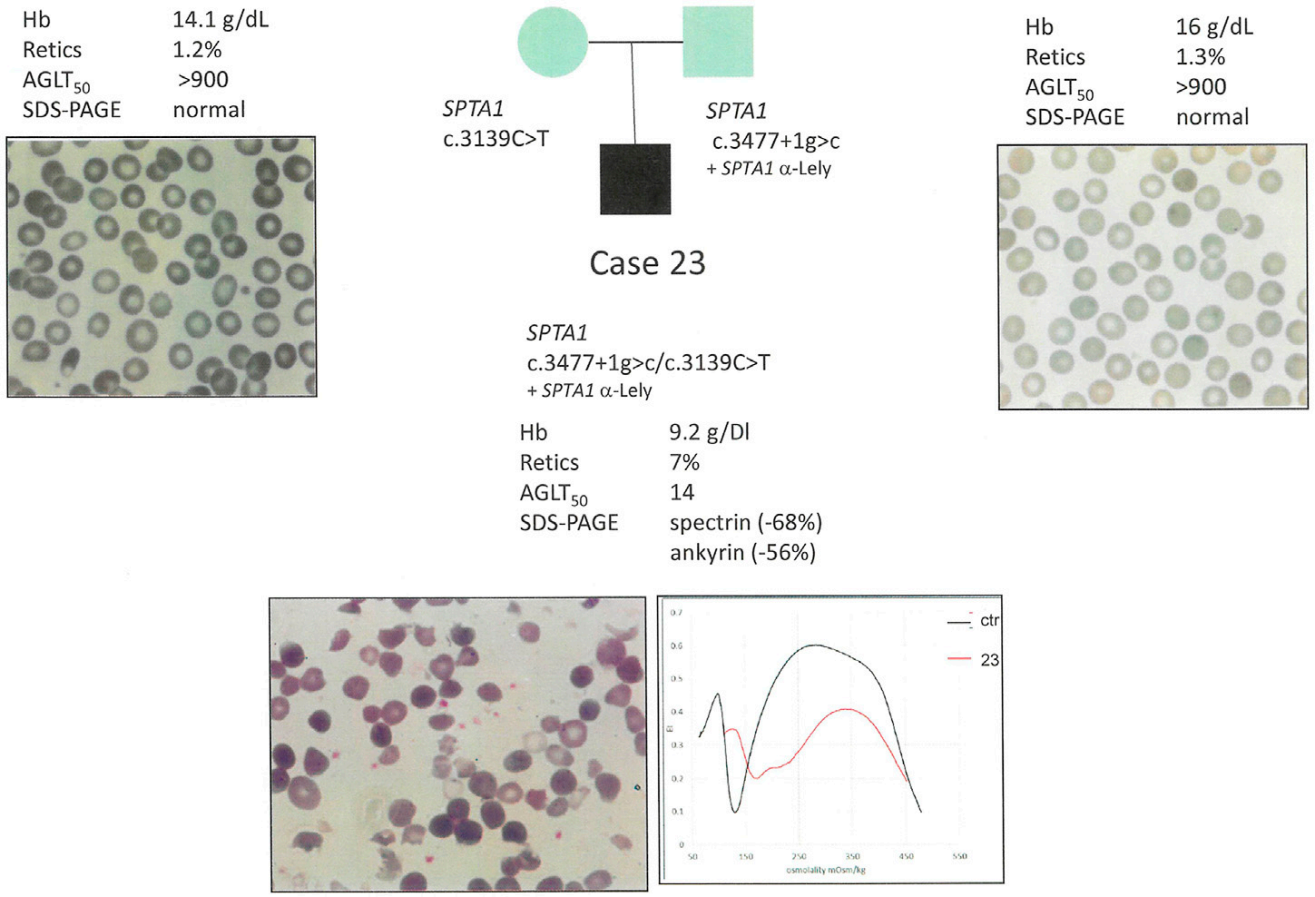
Case 23: Family segregation of the variants, RBC peripheral blood films (100× magnification) and hematological data of the proband and parents.

Conversely, a drastic spectrin defect as observed in case 23 (compound heterozygous for two *SPTA1* disruptive pathogenic variants) with a dramatically compromise RBC morphology (Figure 3) resulted in an additional ankyrin deficiency (-56%).

NGS analysis allowed completing the diagnostic investigations in seven non splenectomized patients with undetectable defect at SDS-PAGE. Of note, most of these cases carried nonsense variants in *SPTB* or *ANK1* genes, confirming a previous report in which splenectomy allowed the detection of spectrin and ankyrin defects as the underlying cytoskeletal abnormality in unclassified HS patients (Miraglia del giudice et al., 1997; Mariani et al., 2008; Vercellati et al., 2022).

Technical reasons may account for the low sensitivity of SDS-PAGE, in fact there are no data available in literature on SDS-PAGE densitometric analysis cut-off limit detection; moreover spectrin and ankyrin content are usually calculated as a ratio on band 3, that is a thick band, and it is possible that slight deficiencies may be lost. Saad et al. (1994) hypothesized that spleen conditioning could peel out some band 3 molecules resulting in overestimation of the other proteins to band 3 ratio. Alternatively, spectrins overexpression may not result in an evident defective incorporation in the RBC membrane (Machnicka et al., 2014). On the other hand SDS-PAGE analysis was able to put in light band 3 deficiency in all cases with *SLC4A1* mutations except one (case 7, p. V308L also resulting in an abnormal splicing), whereas in some cases can reveal protein deficiencies not confirmed by NGS, as in cases 17, 22 and 25. This latter finding is not unexpected since targeted NGS may not identify large deletions due to short DNA sequence read lengths as it has been reported in some cases of *ANK1* or *SPTB* deletions or chromosomal rearrangements (Vermeulen et al., 2002; Panizo Morgado et al., 2020; Wang and Lai 2020).

Finally the concomitant absence of a biochemical and molecular defect in two cases prompted us to re-evaluate the HS diagnosis and their family history.

In case 8 the HS diagnosis was confirmed basing on clinical history and laboratory tests, demonstrating that despite extensive biochemical and molecular investigation, in some cases the first-level diagnostic workflow (faster and cheaper) may have an higher sensitivity than biochemical testing or NGS analysis. On the other hand, as shown in case 24, the HS diagnosis was confirmed on clinical basis despite negative SDS-PAGE and NGS analysis. Positive response to splenectomy and family history of HS, presence of membrane deficiency in the cousin, cholecystectomy/splenomegaly in the family were considered strong enough evidences to confirm the diagnosis.

In conclusion, this study revealed complex relationships between the primary molecular lesion and the final effect in the RBC membrane cytoskeleton. The analysis of some complex/atypical cases demonstrated that there is not a unique approach to the diagnosis of HS; moreover, despite extensive and accurate investigations clinical and family information may still be pivotal in reaching the diagnosis.

## Data Availability

Publicly available datasets were analyzed in this study. This data can be found here: ClinVar SCV001469066-SCV001469086.
